# Identification of serum proteomic biomarkers for early porcine reproductive and respiratory syndrome (PRRS) infection

**DOI:** 10.1186/1477-5956-10-48

**Published:** 2012-08-08

**Authors:** Sem Genini, Thomas Paternoster, Alessia Costa, Sara Botti, Mario Vittorio Luini, Andrea Caprera, Elisabetta Giuffra

**Affiliations:** 1 Parco Tecnologico Padano - CERSA, Via Einstein, 26900, Lodi, Italy; 2 IASMA-FEM Research and Innovation Centre, Via E. Mach 1, 38010, San Michele a/A, TN, Italy; 3 BIOTRACK s.r.l., Via Einstein, 26900, Lodi, Italy; 4 Istituto Zooprofilattico Sperimentale della Lombardia e dell'Emilia Romagna, 26900, Lodi, Italy; 5 Present address: Department of Clinical Studies, School of Veterinary Medicine, University of Pennsylvania, Philadelphia, PA, 19104, USA; 6 Present address: Sandoz Industrial Products S.p.A., Corso Verona 165, 38068, Rovereto, TN, Italy; 7 Present address: INRA, UMR 1313 de Génétique Animale et Biologie Intégrative, Jouy-en-Josas, France

**Keywords:** Porcine reproductive and respiratory syndrome virus (PRRSV), Pig, SELDI-TOF MS, Proteomic fingerprint profiling, Biomarkers, Serum

## Abstract

**Background:**

Porcine reproductive and respiratory syndrome (PRRS) is one of the most significant swine diseases worldwide. Despite its relevance, serum biomarkers associated with early-onset viral infection, when clinical signs are not detectable and the disease is characterized by a weak anti-viral response and persistent infection, have not yet been identified. Surface-enhanced laser desorption ionization time of flight mass spectrometry (SELDI-TOF MS) is a reproducible, accurate, and simple method for the identification of biomarker proteins related to disease in serum. This work describes the SELDI-TOF MS analyses of sera of 60 PRRSV-positive and 60 PRRSV-negative, as measured by PCR, asymptomatic Large White piglets at weaning. Sera with comparable and low content of hemoglobin (< 4.52 μg/mL) were fractionated in 6 different fractions by anion-exchange chromatography and protein profiles in the mass range 1–200 kDa were obtained with the CM10, IMAC30, and H50 surfaces.

**Results:**

A total of 200 significant peaks (p < 0.05) were identified in the initial discovery phase of the study and 47 of them were confirmed in the validation phase. The majority of peaks (42) were up-regulated in PRRSV-positive piglets, while 5 were down-regulated. A panel of 14 discriminatory peaks identified in fraction 1 (pH = 9), on the surface CM10, and acquired at low focus mass provided a serum protein profile diagnostic pattern that enabled to discriminate between PRRSV-positive and -negative piglets with a sensitivity and specificity of 77% and 73%, respectively.

**Conclusions:**

SELDI-TOF MS profiling of sera from PRRSV-positive and PRRSV-negative asymptomatic piglets provided a proteomic signature with large scale diagnostic potential for early identification of PRRSV infection in weaning piglets. Furthermore, SELDI-TOF protein markers represent a refined phenotype of PRRSV infection that might be useful for whole genome association studies.

## Background

Porcine reproductive and respiratory syndrome (PRRS) is one of the most important infectious swine diseases throughout the world [1-3] and is still having, more than two decades after its emergence, major impacts on pig health and welfare (reviewed by [4]). The responsible agent is an enveloped, ca. 15 kb long positive-stranded RNA virus (PRRSV) that belongs to the Arteriviridae family [5] and that can cause late-term abortions in sows and respiratory symptoms and mortality in young or growing pigs. Once this virus has entered a herd it tends to remain present and active indefinitely causing severe economic losses and marketing problems due to high direct medication costs and considerable animal health costs needed to control secondary pathogens [6,7].

Pigs of all ages are susceptible to this highly infectious virus, which has been shown to be present in most pigs for the first 105 days post infection [8]. However clinical manifestations vary with physiological status and age [9], as the virus uses several immune evasion ways to complicate the ability of the host to respond to the infection process [4,10,11]. Weaning piglets, in particular, are likely to be exposed to the infection. Although PRRSV viraemia is often asymptomatic in these piglets, their productive performance is significantly decreased. Indeed, despite being sero-negative, persistently infected piglets still harbor PRRSV and have been shown to be a source of virus for susceptible animals [12].

SELDI-TOF MS analysis allows the comparison of protein profiles obtained from a large number of diverse biological samples by combining two principles, chromatography by retention on chip surface on the basis of defined properties (e.g. charge, surface hydrophobicity, or biospecific interaction with ligands) and mass spectrometry. It is thus distinct from common non-selective techniques, such as two-dimensional polyacrilamide gel electrophoresis (2D-PAGE) and matrix-assisted laser desorption ionisation (MALDI) MS. SELDI-TOF MS has been widely used for diagnostic biomarker discovery and validation across studies in blood serum/plasma, particularly in cancer research (reviewed by [13]), but also to characterize and identify biomarkers associated with viral and other infectious diseases [14-19]. The protein signatures identified by SELDI-TOF MS analysis have thus many potential applications in animal health, including early diagnosis of diseases, prediction of disease states, as well as monitoring of disease progression, recovery, and response to vaccination. Few reports have been published for livestock applications [19-22].

Current needs in veterinary medicine and animal husbandry include the identification of tools that allow the early warning of diseases, especially during the incubation periods and before the onset of clinical signs. Therefore, the objective of this study was to identify by SELDI-TOF MS a proteomic profile able to differentiate PPRSV-positive from -negative weaning piglets raised in commercial farms and without clinical symptoms of the disease. We optimized the experimental conditions previously described [20] and validated 47 statistically significant discriminatory biomarkers. Among these, a combination of 14 biomarkers identified in F1 on CM10 at low focus mass permitted to correctly assign the piglets to the PPRSV-positive or PRRSV-negative groups with sensitivity and specificity of 77% and 73%, respectively.

## Results

To enable identification of medium-low abundant proteins, only samples with a total content of hemoglobin lower than 4.52 μg/mL were included in the study. Total hemoglobin absorbance and the resulting hemoglobin content were calculated for all the piglet sera in both discovery (n = 50) and validation (n = 70) phases of the study [ Additional file 1: Table S1 and Additional file 2: Table S2, respectively].

Fractioning of the sera resulted in six different pH fractions; F1 = pH9, F2 = pH7, F3 = pH5, F4 = pH4, F5 = pH3, and F6 = organic solvent. The fractions F1, F4, and F6 were analyzed on the three surfaces CM10, IMAC30, and H50 at both low and high focus masses. Fractions F2 and F3 were excluded from further analyses because preliminary data with 3 serum samples showed that they still contained elevated quantities of abundant proteins (such as albumin), as well as the quality of the spectra and the number of signals detected were very low. Fraction F5 was excluded because no signals were detected.

The fractions F1, F4, and F6 on the surfaces CM10, IMAC30, and H50 showed generally good signal intensities and low coefficient of variation (CV) values (< 30%) in both the discovery and validation phases. Exceptions were fraction F1 on IMAC30 (analyzed at high focus mass) and H50 (both low and high focus masses), as well as fraction F4 on H50 (low focus mass), which were therefore excluded from further analyses.

### Discovery phase

A total of 50 pig sera, 25 from PRRSV-positive and 25 from PRRSV-negative piglets were analyzed during the discovery phase of the study [ Additional file 1: Table S1].

We found a total of 785 protein peaks in the sera of all samples (Table 1). The most represented pH fraction was F6 (n = 381), followed by F4 (n = 223), and F1 (n = 181). On surface CM10 we identified 317 peaks, on IMAC30 302 peaks, and on H50 166 peaks. Furthermore, a much higher number of peaks (n = 512) was found on low mass range (1–20 kDa) compared to the high (n = 273; 20–200 kDa).

**Table 1:** Protein peaks identified by SELDI-TOF MS in the discovery phase of the study

**Fraction**	**Surface**	**Acquisition focus mass**	**Number of peaks detected**	**Number of significant peaks (p < 0.05)**
1	CM10	Low	67	22
1	CM10	High	56	38
1	IMAC30	Low	58	20
1	IMAC30	High	None, bad signals and CV > 30%	
1	H50	Low	None, bad signals and CV > 30%	
1	H50	High	None, bad signals and CV > 30%	
		**Total F1**	**181**	**80**
4	CM10	Low	51	18
4	CM10	High	37	10
4	IMAC30	Low	73	9
4	IMAC30	High	29	7
4	H50	Low	None, bad signals and CV > 30%	
4	H50	High	33	5
		**Total F4**	**223**	**49**
6	CM10	Low	70	7
6	CM10	High	36	12
6	IMAC30	Low	108	16
6	IMAC30	High	34	6
6	H50	Low	85	18
6	H50	High	48	12
		**Total F6**	**381**	**71**
		**TOTAL**	**785**	**200**

The 785 total number of peaks detected and the 200 statistically significant (p < 0.05) discriminatory peaks associated with PRRS infection that were identified by the Ciphergen Express software are reported with the fraction, the array surface, and the acquisition focus mass (low: 1–20 kDa; high: 20–200 kDa).

Of the total 785 peaks, 200 were statistically significant (p < 0.05) and permitted to discriminate between PRRSV-positive and PRRSV-negative piglets. Discriminatory peaks were found in F1 (n = 80), F4 (n = 49), and F6 (n = 71) on the surfaces CM10 (n = 107), IMAC50 (n = 58), and H50 (n = 35), as well with low (n = 110) and high (n = 90) focus masses (Table 1).

The highest sensitivity (80%) and specificity (76%) were obtained with the 22 discriminatory peaks of F1 on CM10 at low focus mass. Higher sensitivities were found with the 18 peaks of F4 on CM10 at low focus mass (87%), the 7 peaks of F6 on CM10 at low focus mass (85%), and the 12 peaks of F6 on CM10 at high focus mass (87%), however the specificities of these peaks were lower (64%, 66%, and 66%, respectively).

### Validation phase

The validation phase was performed on 35 new PRRSV-positive and 35 new PRRSV-negative piglets using the same experimental conditions applied in the discovery phase [ Additional file 2: Table S2]. Of the total 200 peaks that were significant in the discovery phase, 47 were confirmed in the validation phase (Table 2).

**Table 2:** Discriminatory protein peaks identified in the discovery phase and confirmed in the validation phase

**Fraction**	**Surface**	**Focus mass**	**ROC (regulation)**	**M/Z (kDalton)**	**p-value discovery**	**p-value validation**	**Sensitivity (+/+)**	**Specificity (−/−)**
1	CM10	Low	0.69 (up-regulated)	4.151	0.02	0.00		
1	CM10	Low	0.82 (up-regulated)	4.458	0.00	0.00		
1	CM10	Low	0.32 (down-regulated)	5.468	0.04	0.01		
1	CM10	Low	0.28 (down-regulated)	5.536	0.01	0.01		
1	CM10	Low	0.74 (up-regulated)	8.308	0.00	0.00		
1	CM10	Low	0.71 (up-regulated)	8.516	0.02	0.00		
1	CM10	Low	0.88 (up-regulated)	8.918	0.00	0.00		
1	CM10	Low	0.87 (up-regulated)	9.124	0.00	0.00		
1	CM10	Low	0.82 (up-regulated)	11.404	0.00	0.02		
1	CM10	Low	0.76 (up-regulated)	11.613	0.00	0.02		
1	CM10	Low	0.69 (up-regulated)	13.785	0.02	0.04		
1	CM10	Low	0.80 (up-regulated)	17.218	0.00	0.00		
1	CM10	Low	0.80 (up-regulated)	17.838	0.00	0.00		
1	CM10	Low	0.74 (up-regulated)	19.761	0.00	0.02		
**Total number of significant peaks Fraction 1, CM10, low focus mass: 14**							77%	73%
1	CM10	High	0.82 (up-regulated)	20.322	0.00	0.00		
1	CM10	High	0.77 (up-regulated)	23.496	0.00	0.00		
1	CM10	High	0.76 (up-regulated)	54.107	0.00	0.04		
1	CM10	High	0.76 (up-regulated)	101.410	0.00	0.01		
1	CM10	High	0.76 (up-regulated)	135.096	0.00	0.01		
1	CM10	High	0.87 (up-regulated)	147.351	0.00	0.00		
**Total number of significant peaks Fraction 1, CM10, high focus mass: 6**							58.8%	51.5%
1	IMAC30	Low	0.84 (up-regulated)	4.462	0.00	0.006		
1	IMAC30	Low	0.80 (up-regulated)	8.843	0.00	0.011		
1	IMAC30	Low	0.84 (up-regulated)	8.914	0.00	0.016		
1	IMAC30	Low	0.77 (up-regulated)	8.977	0.001	0.008		
1	IMAC30	Low	0.82 (up-regulated)	9.119	0.00	0.009		
1	IMAC30	Low	0.79 (up-regulated)	9.136	0.00	0.006		
1	IMAC30	Low	0.64 (up-regulated)	11.090	0.056	0.04		
1	IMAC30	Low	0.79 (up-regulated)	17.860	0.00	0.013		
**Total number of significant peaks Fraction 1, IMAC30, low focus mass: 8**							60.6%	51.5%
4	CM10	High	0.70 (up-regulated)	23.162	0.02	0.00		
4	CM10	High	0.67 (up-regulated)	89.049	0.02	0.017		
**Total number of significant peaks Fraction 4, CM10, high focus mass: 2**								
4	IMAC30	High	0.67 (up-regulated)	144.495	0.034	0.00		
**Total number of significant peaks Fraction 4, IMAC30, high focus mass: 1**								
6	CM10	Low	0.76 (up-regulated)	4.161	0.008	0.00		
6	CM10	Low	0.70 (up-regulated)	8.328	0.025	0.013		
6	CM10	Low	0.68 (up-regulated)	8.535	0.041	0.008		
6	CM10	Low	0.70 (up-regulated)	8.552	0.029	0.010		
6	CM10	Low	0.70 (up-regulated)	8.642	0.036	0.013		
6	CM10	Low	0.30 (down-regulated)	14.843	0.010	0.015		
**Total number of significant peaks Fraction 6, CM10, low focus mass: 6**							64.5%	69.7%
6	IMAC30	Low	0.74 (up-regulated)	8.928	0.013	0.029		
6	IMAC30	Low	0.70 (up-regulated)	10.041	0.025	0.025		
6	IMAC30	Low	0.76 (up-regulated)	11.412	0.005	0.00		
6	IMAC30	Low	0.74 (up-regulated)	12.237	0.009	0.002		
6	IMAC30	Low	0.74 (up-regulated)	12.522	0.009	0.004		
6	IMAC30	Low	0.76 (up-regulated)	12.930	0.002	0.003		
6	IMAC30	Low	0.78 (up-regulated)	13.143	0.002	0.004		
6	IMAC30	Low	0.68 (up-regulated)	17.171	0.045	0.018		
**Total number of significant peaks Fraction 6, IMAC30, low focus mass: 8**							54.5%	53%
6	IMAC30	High	0.28 (down-regulated)	27.806	0.023	0.018		
6	IMAC30	High	0.30 (down-regulated)	27.606	0.030	0.017		
**Total number of significant peaks Fraction 6, IMAC30, high focus mass: 2**								

Proteomic features of the 47 discriminatory protein peaks identified by SELDI-TOF MS in the discovery phase and confirmed in the validation phase. The peaks are divided by fraction, array surface, acquisition focus mass (low: 1–20 kDa; high: 20–200 kDa), ROC (Receiver Operating Characteristic = Area Under Curve) value with regulation status in PRRSV-positive compared to PRRSV-negative piglets, molecular weight, and p-values for both discovery and validation phases. The sensitivity and specificity of the total number of discriminatory peaks identified per fraction, array surface and acquisition focus mass is also reported. The sensitivity and specificity were calculated only if the number of peaks was greater than 2.

In particular, 28 peaks were confirmed on CM10, 19 on IMAC30, whereas none of the peaks could be validated on the surface H50. In the 3 fractions with different pH tested, F1 contained 28 peaks, F4 3 peaks, and F6 16 peaks. A higher number of peaks (n = 36) corresponded to small peptides (acquired at low focus mass 1–20 kDa), compared to big peptides (n = 11) that were acquired at high focus mass (20–200 kDa).

**Figure 1:**
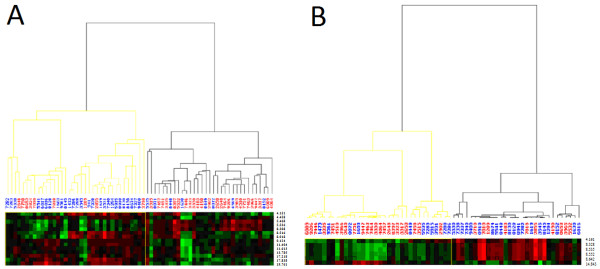
**Heat map showing cluster analysis of the PRRSV-positive and PRRSV-negative piglets tested with the 2 combinations of discriminatory peaks that showed the highest sensitivity and specificity values.** The x-axis of the heat maps shows the piglets analyzed in the validation phase (blue: PRRSV-positive; red: PRRSV-negative), while the y-axis displays the molecular weights in Dalton of the 14 significant discriminatory peaks identified in F1 (**A**) and the 6 peaks in F6 (**B**) both on the surface CM10 at low focus mass. The maps contain peak fold changes Z-score normalized over all piglets. They are color coded, with red corresponding to up-regulation and green to down-regulation in PRRSV-positive piglets. As expected, piglets from the two different groups clustered together, although some incorrectly assigned piglets could be observed (as confirmed by the calculated sensitivities and specificities values, see text).

The vast majority (42) of the peaks were up-regulated in PRRSV-positive piglets compared to the negative, while only 5 peaks (F1 on CM10: 5,468 and 5,536 Da; F6 on CM10: 14,843 Da; and F6 on IMAC30: 27,806 and 27,606 Da) were down-regulated (Table 2). In line with the results of the discovery phase, the combination of peaks with the highest sensitivities (77% and 64.5%) and specificities (73% and 69.7%) were found on CM10 at low focus mass with the 14 discriminatory peaks of F1 and the 6 discriminatory peaks of F6, respectively (Table 2). The correctly and incorrectly assigned piglets using these peaks are graphically illustrated in the heat map of Figure 1; part 1A shows the 14 peaks of F1 and part 1B the 6 peaks identified in F6.

**Figure 2:**
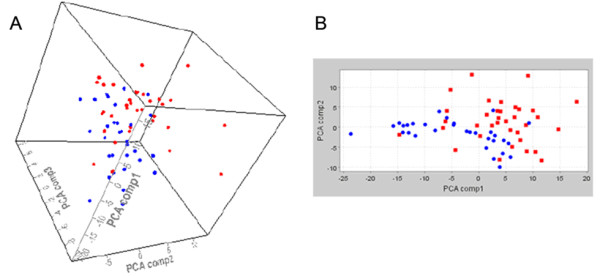
**Principal component analysis (PCA) showing the effects of the 47 significant discriminatory peaks on piglets positive or negative to PRRSV infection.** The figure shows a projection of the measured peak intensities profiles onto the plane spanned by the three principal components (PCAs) that are the axes along which the data vary the most, for the 35 PRRSV-positive (blue) and the 35 PRRSV-negative (red) piglets of the validation study. PCA1, PCA2, and PCA3 accounted for 58.2%, 17.9%, and 12.9% of the variability in the data, respectively. PCA analysis illustrates a 3-dimentional plot comparison of PCA1, PCA2 and PCA3 in the three axes (**A**), as well as 2-dimentional score plot comparisons between PCA1 and PCA2 (**B**).

Principal component analysis (PCA) was performed on the profiles of the 47 discriminatory peaks identified during the discovery and confirmed during the validation phase to identify and quantify independent sources of variation observed in the data. PCA analysis showed that 58.2% (PCA1), 17.9% (PCA2), and 12.9% (PCA3) of the total variability within the data was accounted for the X, Y, and Z axes, respectively. These axes were used to plot the data (Figure 2) and they provide an overview of the variation between the individual samples and show how samples grouped. Figure 2A showed three-dimensionally that the PCA peak profiles of piglets positive to PRRSV differed from piglets negative to PRRSV and revealed a good separation among the profiles of the two different groups, especially considering the high heterogeneity of the samples included in the study, as reported in the MM section and in [ Additional file 1: Table S1 and Additional file 2: Table S2]. Furthermore, with the exception of few outliers, PCA1 combined with PCA2 also separated well the two piglet populations (Figure 2B).

### Comparison with relevant protein peaks and immunity genes related to PRRSV infection in other studies

To provide an overview of the current literature and to try to correlate the discriminatory peaks identified in this study with relevant proteins, we summarized in Table 3 the molecular weights of several peaks that have been shown to be related to PRRSV infection.

**Table 3:** Comparison between relevant PPRSV-related and pig proteins identified in other studies and the discriminatory peaks found in this study

**Method of identification of the peak [reference]**	**Protein**	**Reported MW (kDa)**	**Regulation in other studies**	**MW (kDa) of the peak identified in this study with a difference ≤0.3 kDa compared to the other reports (regulation PRRSV-positive vs. -negative)**
**PRRSV proteins**				
- Calculated molecular mass from amino acid sequence [23,24]	ORF1a – non structural polyprotein	260 - 270		
- Calculated molecular mass from amino acid sequence [23,24]	ORF1ab – non structural polyprotein	420 - 430		
- Estimated size from amino acid sequence [25]	ORF2a - glycoprotein 2a (GP2a)	28.4		
- 2-DE PAGE and MALDI-TOF [26]		29.4		
- SDS page and western of MARC-145 cells infected with PRRSV [27]	ORF2b - non-glycosylated protein 2b	10		10.041 (up-regulated)
- Estimated size from amino acid sequence [25]	ORF3 - glycoprotein 3 (GP3)	30.6		
- 2-DE PAGE and MALDI-TOF [26]		29		
- Estimated size from amino acid sequence [25]	ORF4 - glycoprotein 4 (GP4)	20		19.761 (up-regulated)
- 2-DE PAGE and MALDI-TOF [26]		19.5		19.761 (up-regulated)
- Estimated size from amino acid sequence [25]	ORF5 - glycoprotein 5 (GP5, E)	22.4		
- 2-DE PAGE and MALDI-TOF [26]		22.4		
- Estimated size from amino acid sequence [25]	ORF6 - matrix protein (M)	18.9		
- 2-DE PAGE and MALDI-TOF [26]		19		
- Estimated size from amino acid sequence [25]	ORF7 - nucleocapsid protein (N)	13.8		13.785 (up-regulated)
- 2-DE PAGE and MALDI-TOF [26]		13.5		13.785 (up-regulated)
**Pig protein peaks related to PRRSV infection**				
- MALDI-TOF (sera of pigs after few days of infection with PRRSV vs. normal) [28]	Alpha 1 S (a1S)-subunit of porcine Haptoglobin (Hp)	9.244	Up-regulated in PRRSV infected sera (after 1–7 days)	9.136 (up-regulated)
	Unknown peak	4.165	No difference	4.161 (up-regulated)
	Unknown peak	4.460	No difference	4.458; 4.462 (both up-regulated)
	Unknown peak	5.560	No difference	5.536 (down-regulated)
	Unknown peak	8.330	No difference	8.328 (up-regulated)
	Unknown peak	8.825	No difference	8.843 (up-regulated)
	Unknown peak	12.250/12.55	No difference	12.237/12.522 (both up-regulated)
	Unknown peak	14.010	No difference	13.785 (up-regulated)
- 2-DE PAGE and MALDI-TOF of cellular proteins incorporated in PRRSV virions [26]	Keratin 10	58.8		
	Coronin, actin binding protein, 1B	55.7		
	Keratin 9	62		
	Tubulin, beta polypeptide	47.7		
	Tubulin, alpha, ubiquitous	50.1		
	Beta-actin	41.7		
	Actin, gamma 1 propeptide	41.8		
	Keratin 1	66		
	Tropomyosin 1 alpha chain isoform 4	32.9		
	Cofilin 1 (non-muscle)	18.5		
	Heat shock 70 kDa protein 8 isoform 1	70.8		
	Heat shock 60 kDa protein 1	61		
	Ribosomal protein P0	34.2		
	Heat shock protein 27	22.3		
	Transketolase	67.8		
	Pyruvate kinase	57.8		
	Phosphoglycerate dehydrogenase	56.6		
	Aldehyde dehydrogenase 1A1	54.8		
	UDP-glucose dehydrogenase	55		
	Enolase 1	47.1		
	Phosphoglycerate kinase 1A isoform 2	44.6		
	Glyceraldehyde-3-phosphate dehydrogenase	23.8		23.496 (up-regulated)
	Guanine nucleotide binding protein (G protein), beta polypeptide 1	37.3		
	L-lactate dehydrogenase B	36.6		
	Chain A, Fidarestat Bound To Human Aldose Reductase	35.7		
	PREDICTED: lactate dehydrogenase	36.6		
	Peroxiredoxin 1	22.1		
	Proteasome activator hPA28 subunit beta	27.3		27.606 (down-regulated)
	Triosephosphate isomerase 1	26.6		
	Chaperonin containing TCP1, subunit 3 (gamma)	60.4		
	Chaperonin containing TCP1, subunit 6A (zeta 1)	58		
	Chaperonin containing TCP1, subunit 5 (epsilon) protein	59.6		
	Chaperonin containing TCP1, subunit 2	57.4		
	PRP19/PSO4 pre-mRNA processing factor 19 homolog	55.1		
	Retinoblastoma binding protein 4 isoform a	47.6		
	Eukaryotic translation initiation factor 4A isoform 1	46.1		
	Proliferating cell nuclear antigen	28.7		
	Alpha2-HS glycoprotein	35.6		
	Annexin A2	38.5		
	Annexin A5	35.9		
	Annexin A4	36.1		
	S100 calcium binding protein A10	11.2		11.090 (up-regulated)
	Galectin-1	14.7		14.843 (down-regulated)
	T-complex protein 1 isoform a	60.3		
	Gastric-associated differentially expressed protein YA61P	14.9		14.843 (down-regulated)
- 2-DE PAGE and MALDI-TOF of PAM infected with PRRSV vs. normal [29]	Lymphocyte cytosolic protein 1	70	Up-regulated in infected PAM	
	65 kDa macrophage protein	70.2	Up-regulated	
	L plastin isoform 2	41.4	Up-regulated	
	Enolase 1	47.1	Up-regulated	
	BUB3 budding uninhibited by benzimidazoles 3 isoform a	37.1	Up-regulated	
	Heat shock 27 kDa protein 1	22.9	Up-regulated	23.162 (up-regulated)
	Proteasome beta 2 subunit	22.8	Up-regulated	
	Transgelin 2	21.1	Up-regulated	
	NADP-dependent isocitrate dehydrogenase	46.7	Up-regulated	
	Superoxide dismutase 2	11.7	Up-regulated	11.613 (up-regulated)
	Lamin C	65.1	Up-regulated	
	Aconitase	98.1	Up-regulated	
	Long chain acyl-CoA dehydrogenase	47.9	Up-regulated	
	Proteasome subunit alpha type 1	29.5	Up-regulated	
	70 kDa heat shock cognate protein atpase domain	41.9	Up-regulated	
	Similar to dihydrolipoamide S-succinyltransferase (E2 component of 2-oxo-glutarate complex)	48.9	Up-regulated	
	Similar to cleavage stimulation factor, 3 pre-RNA, subunit 1 isoform 3	47.3	Up-regulated	
	Beta Actin	39.2	Down-regulated in infected PAM	
	Beta Actin	32.1	Down-regulated	
	Myoglobin	16.9	Down-regulated	17.171 (up-regulated)
	Vacuolar protein sorting 29	20.5	Down-regulated	20.322 (up-regulated)
	Transketolase	67.9	Down-regulated	
	Eukaryotic translation initiation factor 3, subunit 5	37	Down-regulated	
	Cathepsin D protein	42.7	Down-regulated	
	Similar to lymphocyte-specific protein 1	40.9	Down-regulated	
- 2-DE PAGE and MALDI-TOF of PAM constitutively expressing the PRRSVN protein vs. normal [30]	Proteasome subunit alpha type 6	28.5	Up-regulated in PAM expressing PRRSVN	
	Heat shock protein 27 kDa	23	Up-regulated	23.162 (up-regulated)
	Annexin 1	38.5	Up-regulated	
	Septin 2	42.9	Up-regulated	
	Spermidine synthase	34.4	Down-regulated in PAM expressing PRRSVN	
	Major vault protein	19.3	Down-regulated	
	Ferritin L subunit	18.3	Down-regulated	
	Nucleoside diphosphate kinase A	17.3	Down-regulated	17.218 (up-regulated)
	Chaperonin containing TCP-1 beta subunit	57.8	Down-regulated	
	Dihydropyrimidinase related protein 2	62.7	Down-regulated	
	Translation elongation factor 2	47.2	Down-regulated	
- 2-DE PAGE and MALDI-TOF of PAM and Marc-145 cells infected with PRRSV [31]	Cofilin 1	25.773	Up-regulated in Marc-145	
	Actin-related protein	16.278	Up-regulated in PAM	
	Vimentin	30.826	Up-regulated in PAM	
	Alpha cardiac actin	16.758	Up-regulated in PAM	
	Cofilin 1	18.507	Up-regulated in PAM	
	Stress 70 protein	55.119	Up-regulated in Marc-145	
	Peroxiredoxin 2	19.418	Up-regulated in Marc-145	
	Heat shock 27 kDa protein 1	22.927	Up-regulated in Marc-145	23.162 (up-regulated)
	Peroxiredoxin 6	24.995	Up-regulated in Marc-145	
	Heat shock protein beta 1 (HSPB1)	22.768	Up-regulated in PAM	
	Ubiquitin	8.559	Up-regulated in Marc-145	8.552 (up-regulated)
	Cystatin B (CSTB)	25.288	Up-regulated in PAM	
	FYVE finger containing phosphoinositide kinase	232.904	Up-regulated in PAM	
	Pyruvate kinase isozymes M1/M2 (PKM2)	57.744	Up-regulated in PAM	
	UPF 0681 protein KIAA1033	136.330	Up-regulated in Marc-145	
	Tropomyosin alpha 4 chain (TPM4)	28.504	Up-regulated in PAM	
	UPF 0568 protein	28.191	Up-regulated in PAM	
	LIM and SH3 protein 1	29.975	Down-regulated in Marc-145	
	Plectin 1	532.578	Down-regulated in Marc-145	
	Glial fibrillary acidic protein (GFAP)	46.497	Down-regulated in Marc-145	
	Plectin 1	516.572	Down-regulated in PAM	
	Galectin 1	14.736	Down-regulated in Marc-145	14.843 (down-regulated)
	Galectin 1	14.590	Down-regulated in PAM	
	Superoxide dismutase 1 (SOD1)	15.236	Down-regulated in PAM	
	Prohibitin	29.757	Down-regulated in Marc-145	
	Epidermal fatty acid-binding protein 5 (FABP5)	15.199	Down-regulated in PAM	
	A kinase anchoring protein AKAP350	416.855	Down-regulated in PAM	
	Pyridoxine 5 phosphate oxidase variant	29.896	Down-regulated in PAM	

List of relevant PPRSV and pig proteins that have been shown in other studies and might correspond to the significantly expressed peaks found here with SELDI-TOF MS. The method used in other studies to identify the peak with the corresponding reference, the protein names, as well as their MW and regulation are reported. The last column indicates the MWs (with in parenthesis the regulation in PRRSV-positive compared to PRRSV-negative piglets) of discriminatory peaks identified in this study that showed a difference ≤0.3 kDa compared to the other studies

First of all, we summarized the available information on the PRRS viral proteins. The PRRSV genome is ca. 15 kb in size and consists of the 5' untranslated region (UTR), at least nine open reading frames (ORFs), and the 3' UTR followed by a polyadenylation tail. The expected and experimentally identified MWs for each viral protein from different studies are reported in Table 3, along with the MW of the closest discriminatory peak identified in the current study.

Interestingly, the MW of the viral proteins ORF2b, ORF4, and ORF7 were very similar (difference of MW ≤0.3 kDa) to up-regulated discriminatory peaks identified here (Table 3).

As next, we compared proteins related to PRRSV infection that were identified in additional studies (Table 3); interestingly, all the 9 peaks found by [28], and in particular the only up-regulated in PRRSV infected (corresponding to the Alpha 1 S (a1S)-subunit of porcine Haptoglobin), showed minimal MW differences (≤0.3 kDa) with up-regulated peaks identified in this study (Table 3).

Additional discriminatory peaks found in the current study were very similar (MW differences ≤0.3 kDa) to those identified in other PRRS-related proteomic studies (Table 3). They corresponded to the following proteins: Glyceraldehyde-3-phosphate dehydrogenase, Proteasome activator hPA28 subunit beta, S100 calcium binding protein A10, Galectin 1, and Gastric-associated differentially expressed protein YA61P [26]; Heat shock 27 kDa protein 1, Superoxide dismutase 2, Myoglobin, and Vacuolar protein sorting 29 [29]; Heat shock protein 27 kDa and Nucleoside diphosphate kinase A [30]; Heat shock 27 kDa protein 1, Galectin 1, and Ubiquitin [31].

## Discussion

In the present work, we show that proteomic fingerprint profiling is useful in researches on PRRS immuno-pathogenesis and might also be a robust, large scale diagnostic tool for the assessment of the proportion of PRRSV-positive weaning piglets without clinical symptoms in a herd. Indeed, we confirmed that the high-throughput capacity of the SELDI-TOF MS technology allows the screening for disease biomarkers of hundred of samples in a relative short-time period and with minimal sample preparation (as previously also reported by [32]).

Our results indicate that from the 200 significant peaks found in the discovery phase, a total of 47 could be confirmed in the validation phase. These values are comparable with another study where similar experimental conditions were applied to ovine sera [19].

Our findings also show that the combination of 14 discriminatory peaks in F1 on CM10 at low focus mass provided the highest sensitivity of 77% and specificity of 73% to correctly assign the piglets to the PPRSV-positive or PRRSV-negative groups. These percentages are in line with recent studies in humans using the same technology [33,34]. Also the PCA results showed a good separation of the piglets in the two groups under examination. This was reached even though the tested piglets had large variability and heterogeneity, as they were collected from several farms located in different regions, and underwent high environmental pressures, typical of the field conditions. This is mainly due to the careful choice of the serum samples, where we tried to minimize the environmental differences by using same experimental parameters (e.g. sample collection procedures, storage, handling) and by including a similar number of pigs from the same breed (Large White) and with very similar sex ratios and ages (at weaning).

In a preliminary work [20] we had successfully transferred the experimental conditions used in profiling experiments of human sera to pig sera. However, in that work, none of the potential biomarkers identified in the discovery phase could be validated in the subsequent validation phase, because of high samples heterogeneity and high content of serum (e.g. albumin) and contaminant proteins (e.g. hemoglobin), having a negative effects on the detection of significant biomarkers, particularly those corresponding to the medium-low abundant proteins. It has been reported that low abundant proteins constitute about 1% of the entire human serum proteome, with the remaining 99% being comprised of only 22 proteins [35]. As it was therefore necessary to reduce the level of abundant proteins, in this follow up study, particular relevance was given to the content of the contaminant protein hemoglobin. Only non-hemolytic samples with similar, low contents of hemoglobin were included in the study. Additionally, to further increase the likelihood to identify statistically significant discriminatory biomarkers, we introduced a fractioning step based on anion-exchange chromatography. In a similar study performed with MALDI-TOF [28], where serum samples were analyzed in the first weeks (2–16) of PRRSV infection (also verified by PCR), a significantly lower number of peaks were identified compared to the present work. While protein peaks with M/Z values of 4.165, 4.460, 5.560, 8.330, 8.825, 12.250/12.550, and 14.010 kDa were found in 94 serum samples from 59 pigs, only one peak (9.244 kDa), corresponding to the alpha 1 S (a1S)-subunit of porcine Haptoglobin (Hp), was differentially up-regulated in PRRSV infected pigs. Interestingly, all these peaks were very similar (MW difference ≤0.3 kDa) with discriminatory peaks identified here (details in Table 3). Furthermore, two peaks identified in this study (23.162 and 14.843 kDa) were similar to peaks identified elsewhere (corresponding to Heat shock 27 kDa protein 1 [29-31] and Galectin 1 [26,31], respectively). In accordance with [31], the identified peak corresponding to Heat shock 27 kDa protein 1 was up-regulated, while the peak corresponding to Galectin 1 was down-regulated. Thus, these proteins seem to be very interesting and suitable candidates for future investigations.

The preponderance of the significant biomarkers had a molecular mass lower than 20 kDa, confirming that small peptides are a rich source of relevant biomarkers in SELDI-TOF MS analyses as previously reported in human [36] and ovine [19] sera. This may also partly be caused by the fact that the low molecular weight region (LMW) of the serum proteome, called peptidome, is an assortment of small intact proteins and proteolytic fragments of larger proteins, including several classes of physiologically important proteins like peptide hormones and components of both the innate and adaptive immune systems (i.e. cytokines and chemokines) [35,37]. This is particularly interesting as the patho-physiological state of the body’s tissue is predominantly reflected in the LMW and low abundance region of the serum proteome, and specific protein fragments of the serum peptidome have been shown to contain a rich source of disease-specific diagnostic information and they have been correlated with disease stages in several studies (reviewed by [37]).

In agreement with other studies [29,31], we found that the majority of the discriminatory biomarkers were up-regulated in PRRSV-positive piglets. This seems to suggest that the corresponding proteins might be of viral origin or related to the innate or adaptive immune responses (e.g. cytokines, chemokines, acute phase proteins, toll like receptors). In fact, several peaks showed high similarities (MW differences ≤0.3 kDa) with previous works, in particular regarding viral proteins (Table 3). The assignment of the discriminatory peak to a specific protein will require additional work, because the SELDI-TOF technology can only detect masses/peaks of proteins that are differentially expressed between samples but can not directly identify the proteins. This represents one of the major drawbacks of this technology compared to other methods. However, an advantage of the SELDI-TOF MS in this regard is that the results of this technique might lead to the identification of new proteins that were previously not correlated to the disease, and this might hopefully lead to the identification of new biomarkers representing the field situation. The interpretation of these results and the continuation of this project will benefit from the very imminent termination and publication of the sequence of the swine genome [38], which will definitely contribute to a more precise annotation and a better identification of genes and proteins and thus will greatly facilitate genome wide mapping association studies.

## Conclusions

Although a combination of peaks identified with different experimental conditions (e.g. using different fractions and different surfaces) might have provided higher discriminatory power, here we developed a PRRSV diagnostic test based on peaks identified with the same experimental conditions (e.g. fraction, surface, and focus mass), which can be reproduced at high-throughput at reasonable costs. These results provide a set of proteomic biomarkers and related, optimized experimental conditions for high-throughput profiling of pig populations by SELDI-TOF MS for whole genome association studies, where identification of proteins underlying the phenotype can be made *a posteriori*. SELDI-TOF MS might therefore represent a complementary test or a possible alternative to classical (PCR) and more recent diagnostic methods (e.g. antibody detection in saliva) for profiling large flocks of pigs at reasonable costs, using blood samples that are routinely collected for general veterinary inspections. As well, these SELDI-TOF MS based tests could complement and provide a broader reference for emerging diagnostic methods and have potential applications for the detection of relevant proteins having highly heritable traits (e.g. acute phase proteins).

## Methods

### Piglets

A total of 120 serum samples of Large White piglets were selected from a well defined and characterized repository database, presently containing more than 20,000 swine samples from 18 different farms of the Lombardy region, Italy. Selection of the piglets aimed to minimize environmental factors and experimental conditions that might influence the results [39]. Hence, all piglets were from the same breed (Large White), had similar ages (weaning: 45–50 days), and their sera showed a low and comparable amount of hemoglobin (calculated as shown below).

In the discovery phase of the study, a total of 50 pig sera, 25 from PRRSV-positive and 25 from PRRSV-negative piglets, as determined by PCR (see below), were analyzed [ Additional file 1: Table S1]. The validation phase was performed with the same experimental conditions as the discovery phase. A total of 35 new PRRSV-positive and 35 new PRRSV-negative piglets were examined [ Additional file 2: Table S2]. The actual duration of infection for each individual PRRSV-positive piglet was unknown, as sera were collected and analyzed once for each piglet (at weaning: 45–50 days of age). None of the piglets was treated, as they did not show any symptom of the disease.

To ensure large variability and heterogeneity of the samples and minimize environmental differences, we included in the PRRSV-positive and -negative groups similar numbers of piglets with the same sex that originated from several farms located in different regions. In fact, PRRSV-positive piglets originated from 6 farms of the Lodi region (n = 8) and 7 farms of the Mantua region (n = 52), while PRRSV-negative piglets were collected in 5 farms around Lodi (n = 19) and 9 farms around Mantua (n = 41). Sex ratios males/females (44/76) were very similar in PRRSV-positive (21 vs. 39) and -negative (23 vs. 37) piglets, respectively.

Veterinary inspections of the overall clinical status of the piglets at the day of serum collection did not evidence any clinical symptoms of PRRS, including respiratory distress or sneezing.

### Serum samples

All the serum samples were collected, stored, and handled in the same way. They were obtained for each piglet by storing two mL of whole blood without anticoagulants at room temperature (RT) for 4 h followed by centrifugation at 3,500 rpm for 4 min. As suggested in a previous work [20], an abundant quantity of hemoglobin in the serum can hide early diagnostic biomarkers of PRRSV by competing with the other serum components for the binding site of the chromatographic surfaces. To avoid the consequent signal suppression of the medium-low abundant proteins, only non-hemolytic samples were included in the present study.

A total of 200 clear, transparent sera without red pigmentation (low hemoglobin content) were first selected by visual screening from the total sera available in the database. Hemoglobin content of each serum sample was then determined according to [40] with minor modifications. A calibration curve was generated using five standard solutions (concentrations: 1.8, 3.6, 5.4, 7.2, and 9 μg/ml) of porcine hemoglobin diluted in 400 μL commercially available porcine serum (Sigma Aldrich, St Louis, MO, USA). Triplicate samples were incubated for 5Â·min at RT, then absorbance (E) was measured at 380, 415, and 440 nm. Absorbance at 380 and 440 nm was used to discern background absorbance flanking the absorbance peak (415Â·nm) of oxygenated hemoglobin. Absorbance due to hemoglobin was calculated as: E415–[(E380 + E440)/2]. Hemoglobin absorbance values of the samples were converted to μg/mL of hemoglobin by means of the calibration curve. Of the 200 initial samples, a total of 120 samples having an absorbance ≤ 0.085 (corresponding to a hemoglobin content below 4.52 μg/mL) were included in the study; 50 in the discovery and 70 in the validation phases, respectively.

Viral RNA extraction from the sera was performed following standard Roche procedures (High Pure Viral RNA Kit, Roche Diagnostics GmbH, Germany). Presence or absence of PRRSV was determined by multiplex PCR of conserved regions of viral ORF7 using primers and conditions previously described [41,42]. The test also enabled to discriminate European and American genotypes and could detect all the different viral strains present in the Lombardy region at the time of sample collection.

### Serum fractionation

All the detailed steps of the SELDI-TOF MS process performed here are schematically represented [see Additional file 3: Figure S1]. The protocol follows the manufacturer’s instructions with minor modifications (Bio-Rad Laboratories, ProteinChip® Serum Fractionation Kit manual).

Briefly, serum samples were pre-fractionated with U9 buffer (9 M urea, 2% 3-[(3-Cholamidopropyl)dimethylammonio]-1-propanesulfonate (CHAPS), 50 mM Tris–HCl, pH = 9) to favor dissociation of protein complexes [ Additional file 3: Figure S1A].

Sera were fractionated using a ProteinChip Q strong anion-exchange resin filtration plate (Bio-Rad Laboratories, Hercules, CA). The filtration plate was re-hydrated and equilibrated with rehydration buffer (50 mM Tris–HCl, pH = 9) and the resin washed with rehydration buffer and U1 solution (1 M urea, 0.2% CHAPS, 50 mM Tris–HCl, pH = 9) [ Additional file 3: Figure S1B]. Serum samples were then mixed with U1 solution and added to the equilibrated filtration plate. Successive elutions with different buffers with decreasing pH and a final organic solvent (= different fractions) were collected by centrifugation. The buffers used included pH = 9 (50 mM Tris–HCl, 0.1% n-octyl β-D-glucopyranoside (OGP)), pH = 7 (50 mM 4-(2-Hydroxyethyl)piperazine-1-ethanesulfonic acid (HEPES), 0.1% OGP), pH = 5 (100 mM Na acetate, 0.1% OGP), pH = 4 (100 mM Na acetate, 0.1% OGP), pH = 3 (100 mM Na citrate, 0.1% OGP), and organic solvent (33.3% isopropanol, 16.7% acetonitrile, 0.1% trifluoroacetic acid) [ Additional file 3: Figure S1C].

### ProteinChip arrays

The six pH fractions obtained (F1 = pH9, F2 = pH7, F3 = pH5, F4 = pH4, F5 = pH3, and F6 = organic solvent) were profiled on weak cation-exchange (CM10), immobilized metal affinity capture-copper (IMAC30-CU), and reverse-phase (H50) ProteinChip® arrays. The arrays were initially placed in a Bioprocessor (C50-30011, Bio-Rad Laboratories) and then treated according to their surface [ Additional file 3: Figure S1D]. Each sample fraction was then bound/spotted randomly to the different ProteinChip® arrays using array-specific binding buffers [ Additional file 3: Figure S1E]. A 50% saturated sinapinic acid (SPA) matrix solution was finally added to each spot on the ProteinChip array prior to the final analysis [ Additional file 3: Figure S1F].

### SELDI-TOF MS analysis

ProteinChip arrays were read using a Ciphergen Protein-Chip Reader PCS4000 model and data were analyzed with Ciphergen Express Software (Ciphergen Biosystems).

Profiles were collected in the range 1–200 kDa at the two different ion focus mass 10 kDa (“low focus mass”) and 50 kDa (“high focus mass”). The instrument was calibrated for dataset collection using all-in-one peptide standard (Bio-Rad Laboratories) in the 1–20 kDa range for 10 kDa low ion focus mass and all-in-one protein standard in the 20–200 kDa range for 50 kDa high ion focus mass [ Additional file 3: Figure S1G].

### Ciphergen Express software analysis

Spectra were normalized by total ion current, starting and ending at the M/Z of the collection ranges (1–20 or 20–200 kDa) after baseline subtraction and noise calculation. Outlier spectra were removed. The spectra were aligned to a reference spectrum with the normalization factor nearest 1.0. The spectra were aligned only if the percentage coefficient of variation was reduced after the alignment. Peaks from the different spectra were aligned using the cluster wizard function of the Ciphergen Express 3.0.6 software. The peak detection was automated within the M/Z range of analysis. Peaks were detected on the first pass when the signal-to-noise (S/N) ratio was 7 and the peak was 5 times the valley depth. Peaks below threshold were deleted and all first-pass peaks were preserved. Clusters were created within 0.15% of M/Z for each peak detected in the first pass. The clusters were completed by adding peaks with S/N ratio of 2 and two times the valley depth. P-values and ROC/AUC (Receiver Operating Characteristic/ Area Under Curve) values were calculated by using the P-value wizard.

A 2-tailed t-test was used for statistical analysis of differences in peak intensity between groups. P-values below 0.05 were considered statistically significant. Principal component analysis (PCA) and agglomerative hierarchical clustering algorithm were applied to investigate the pattern among the different statistically significant peaks.

PCA is a multivariate data analysis that transforms without a loss of essential information a number of correlated variables into a smaller number of uncorrelated variables called principal components (PCs), which can explain sufficiently the data structure. PCA transformation allows studying many variables simultaneously, showing how similar samples are correlated and grouped together. The data structure is visualized directly in a graphical way by projection of objects onto the space defined by the selected PCAs (for details see [43]).

Finally, to evaluate the influence of external variables (e.g. sample processing and acquisition) on the system under study and to calculate the dispersion of the acquired data, the coefficient of variation (CV), which is the normalized measure of dispersion of a probability distribution and shows the% dispersion of the data in rapport to the media (intensity variation), was also calculated. Six serum samples commercially available were prepared and analyzed in parallel with the pig samples of both, discovery and validation phases. The CV was calculated for all fractions and surfaces by choosing 6 peaks evenly distributed along the entire range.

## Competing interests

The authors declare that they have no competing interests.

## Authors’ contributions

SG established the experimental settings, prepared the samples, helped conceiving the study design, and wrote the manuscript. TP helped with the analysis of the data and the manuscript writing. ACO conducted the SELDI-TOF MS experiment and performed statistical analyses. SB arranged for sample collection and helped to draft the manuscript. MVL developed the primers to detect PRRSV and performed the PCR analyses to assess presence/absence of the virus. ACA was responsible for data storage and database maintenance. EG coordinated the overall project and participated in the design of the study and helped to draft the manuscript. All authors read and approved the final manuscript.

## Supplementary Material

Additional file 1 **Table S1.**Pigs tested with SELDI-TOF MS during the discovery phase of the study. List of the 25 positive and 25 negative pigs to PRRS (PCR-tested) analyzed with SELDI-TOF MS during the discovery phase of the study. The pig ID is reported with the total absorbance and the total amount of hemoglobin present in the sample, the status regarding the PRRS virus, as well as the sex and the number and location of the farm (MA = Mantua region, LO = Lodi region). Click here for file

Additional file 2 **Table S2.**Pigs tested with SELDI-TOF MS during the validation phase of the study. List of the 35 positive and 35 negative pigs to PRRS (PCR-tested) analyzed with SELDI-TOF MS during the validation phase of the study. The pig ID is reported with the total absorbance and the total amount of hemoglobin present in the sample, the status regarding the PRRS virus, as well as the sex and the number and location of the farm (MA = Mantua region, LO = Lodi region). Click here for file

Additional file 3 **Figure S1.**Detailed protocol of the SELDI-TOF MS analysis. Schematic illustration of the protocol used for SELDI-TOF MS, which follows the manufacturer’s instruction manual with minor modifications (Bio-Rad Laboratories, ProteinChip® Serum Fractionation Kit manual). The protocol is divided in 7 main steps: **A**) Pre-fractionation of the sera; **B**) Rehydration and equilibration of the Protein Chip Q strong anion-exchange resin filtration plate; **C**) Fractionation of the sera; **D**) Preparation of ProteinChip® Arrays; **E**) Binding of the serum fractions to the arrays; **F**) Preparation and application of the matrix; and **G**) SELDI-TOF MS analysis. Click here for file
