# Supplementary material for: Identification of serum proteomic biomarkers for early porcine reproductive and respiratory syndrome (PRRS) infection
Source: Proteome Sci. 2012 Aug 8;10:48. doi: 10.1186/1477-5956-10-48 (PMC3492009; doi:10.1186/1477-5956-10-48)
Supplement: Additional file 2 — Table S2.Pigs tested with SELDI-TOF MS during the validation phase of the study. List of the 35 positive and 35 negative pigs to PRRS (PCR-tested) analyzed with SELDI-TOF MS during the validation phase of the study. The pig ID is reported with the total absorbance and the total amount of hemoglobin present in the sample, the status regarding the PRRS virus, as well as the sex and the number and location of the farm (MA = Mantua region, LO = Lodi region). [file 1477-5956-10-48-S2.doc]

**Additional file 2: Pigs tested with SELDI-TOF MS during the validation phase of the study.**

List of the 35 positive and 35 negative pigs to PRRS (PCR-tested) analyzed with SELDI-TOF MS during the validation phase of the study. The pig ID is reported with the total absorbance and the total amount of hemoglobin present in the sample, the status regarding the PRRS virus, as well as the sex and the number and location of the farm (MA = Mantua region, LO = Lodi region).

| **Serum number** | **Total absorbance** | **Hemoglobin content (**µg/mL) | **PRRSV (PCR tested)** | **Sex** | **Farm # (Location)** | **Serum number** | **Total absorbance** | **Hemoglobin content (**µg/mL) | **PRRSV (PCR tested)** | **Sex** | **Farm # (Location)** |
| --- | --- | --- | --- | --- | --- | --- | --- | --- | --- | --- | --- |
| **0093** | 0.058 | 3.68 | positive | M | 106 (LO) | **0653** | 0.067 | 2.63 | negative | F | 107 (MA) |
| **0941** | 0.07 | 2.95 | positive | F | 108 (MA) | **1289** | 0.03 | undetectable | negative | M | 107 (MA) |
| **1294** | 0.055 | 1.38 | positive | F | 107 (MA) | **2640** | 0.071 | 3.05 | negative | M | 112 (LO) |
| **1473** | 0.069 | 2.84 | positive | M | 112 (LO) | **2648** | 0.085 | 4.52 | negative | F | 112 (LO) |
| **1603** | 0.084 | 4.41 | positive | F | 107 (MA) | **2650** | 0.079 | 3.89 | negative | M | 112 (LO) |
| **1619** | 0.069 | 2.84 | positive | M | 109 (MA) | **2855** | 0.049 | 0.75 | negative | F | 103 (LO) |
| **1630** | 0.036 | undetectable | positive | F | 109 (MA) | **2902** | 0.069 | 2.84 | negative | F | 107 (MA) |
| **2451** | 0.078 | 3.78 | positive | F | 107 (MA) | **3317** | 0.06 | 1.90 | negative | F | 113 (MA) |
| **3531** | 0.059 | 1.80 | positive | M | 107 (MA) | **3323** | 0.046 | 0.44 | negative | F | 113 (MA) |
| **3545** | 0.044 | 0.23 | positive | M | 107 (MA) | **3330** | 0.039 | undetectable | negative | M | 113 (MA) |
| **4569** | 0.043 | 0.12 | positive | M | 107 (MA) | **3335** | 0.043 | 0.12 | negative | F | 113 (MA) |
| **5330** | 0.075 | 3.46 | positive | F | 102 (LO) | **3515** | 0.075 | 3.47 | negative | F | 112 (LO) |
| **5535** | 0.058 | 1.69 | positive | M | 110 (MA) | **3710** | 0.049 | 0.75 | negative | F | 113 (MA) |
| **5541** | 0.058 | 1.69 | positive | F | 110 (MA) | **3824** | 0.055 | 1.38 | negative | M | 107 (MA) |
| **5915** | 0.064 | 2.32 | positive | F | 110 (MA) | **3965** | 0.057 | 1.59 | negative | M | 103 (LO) |
| **6816** | 0.042 | 0.02 | positive | F | 110 (MA) | **3966** | 0.08 | 3.99 | negative | F | 103 (LO) |
| **6817** | 0.068 | 2.74 | positive | F | 110 (MA) | **4064** | 0.068 | 2.74 | negative | M | 107 (MA) |
| **7282** | 0.049 | 0.75 | positive | F | 110 (MA) | **4183** | 0.045 | 0.33 | negative | F | 110 (MA) |
| **7283** | 0.043 | 0.12 | positive | F | 110 (MA) | **4190** | 0.054 | 1.27 | negative | F | 110 (MA) |
| **7286** | 0.083 | 4.31 | positive | F | 110 (MA) | **4510** | 0.075 | 3.47 | negative | M | 110 (MA) |
| **7288** | 0.052 | 1.06 | positive | F | 110 (MA) | **5532** | 0.075 | 3.47 | negative | F | 110 (MA) |
| **7337** | 0.063 | 2.21 | positive | M | 107 (MA) | **6089** | 0.069 | 2.84 | negative | M | 112 (LO) |
| **7338** | 0.054 | 1.27 | positive | F | 107 (MA) | **7010** | 0.071 | 3.05 | negative | M | 112 (LO) |
| **7340** | 0.076 | 3.57 | positive | M | 107 (MA) | **7446** | 0.046 | 0.44 | negative | F | 113 (MA) |
| **8071** | 0.055 | 1.38 | positive | F | 107 (MA) | **7451** | 0.07 | 2.95 | negative | F | 113 (MA) |
| **8072** | 0.056 | 1.48 | positive | F | 107 (MA) | **7457** | 0.068 | 2.74 | negative | F | 113 (MA) |
| **8128** | 0.044 | 0.23 | positive | F | 107 (MA) | **7458** | 0.081 | 4.10 | negative | F | 113 (MA) |
| **8130** | 0.079 | 3.89 | positive | F | 107 (MA) | **7463** | 0.052 | 1.06 | negative | F | 113 (MA) |
| **8145** | 0.053 | 1.17 | positive | F | 107 (MA) | **7464** | 0.059 | 1.80 | negative | F | 113 (MA) |
| **8152** | 0.076 | 3.57 | positive | F | 102 (LO) | **7750** | 0.068 | 2.74 | negative | M | 111 (MA) |
| **8448** | 0.054 | 1.27 | positive | F | 110 (MA) | **7751** | 0.077 | 3.69 | negative | M | 111 (MA) |
| **8449** | 0.051 | 0.96 | positive | M | 110 (MA) | **7818** | 0.058 | 3.68 | negative | F | 103 (LO) |
| **8674** | 0.049 | 0.75 | positive | F | 106 (LO) | **8397** | 0.06 | 1.90 | negative | F | 101 (LO) |
| **9400** | 0.071 | 3.05 | positive | M | 106 (LO) | **9206** | 0.063 | 2.21 | negative | F | 101 (LO) |
| **9561** | 0.059 | 1.80 | positive | M | 105 (LO) | **9447** | 0.062 | 2.11 | negative | M | 112 (LO) |
