# Supplementary material for: Identification of serum proteomic biomarkers for early porcine reproductive and respiratory syndrome (PRRS) infection
Source: Proteome Sci. 2012 Aug 8;10:48. doi: 10.1186/1477-5956-10-48 (PMC3492009; doi:10.1186/1477-5956-10-48)

**Additional file 3: Detailed protocol of the SELDI-TOF MS analysis.**

Schematic illustration of the protocol used for SELDI-TOF MS, which follows the manufacturer’s instruction manual with minor modifications (Bio-Rad Laboratories, ProteinChip® Serum Fractionation Kit manual). The protocol is divided in 7 main steps: **A**) Pre-fractionation of the sera; **B**) Rehydration and equilibration of the Protein Chip Q strong anion-exchange resin filtration plate; **C**) Fractionation of the sera; **D**) Preparation of ProteinChip® Arrays; **E**) Binding of the serum fractions to the arrays; **F**) Preparation and application of the matrix; and **G**) SELDI-TOF MS analysis.


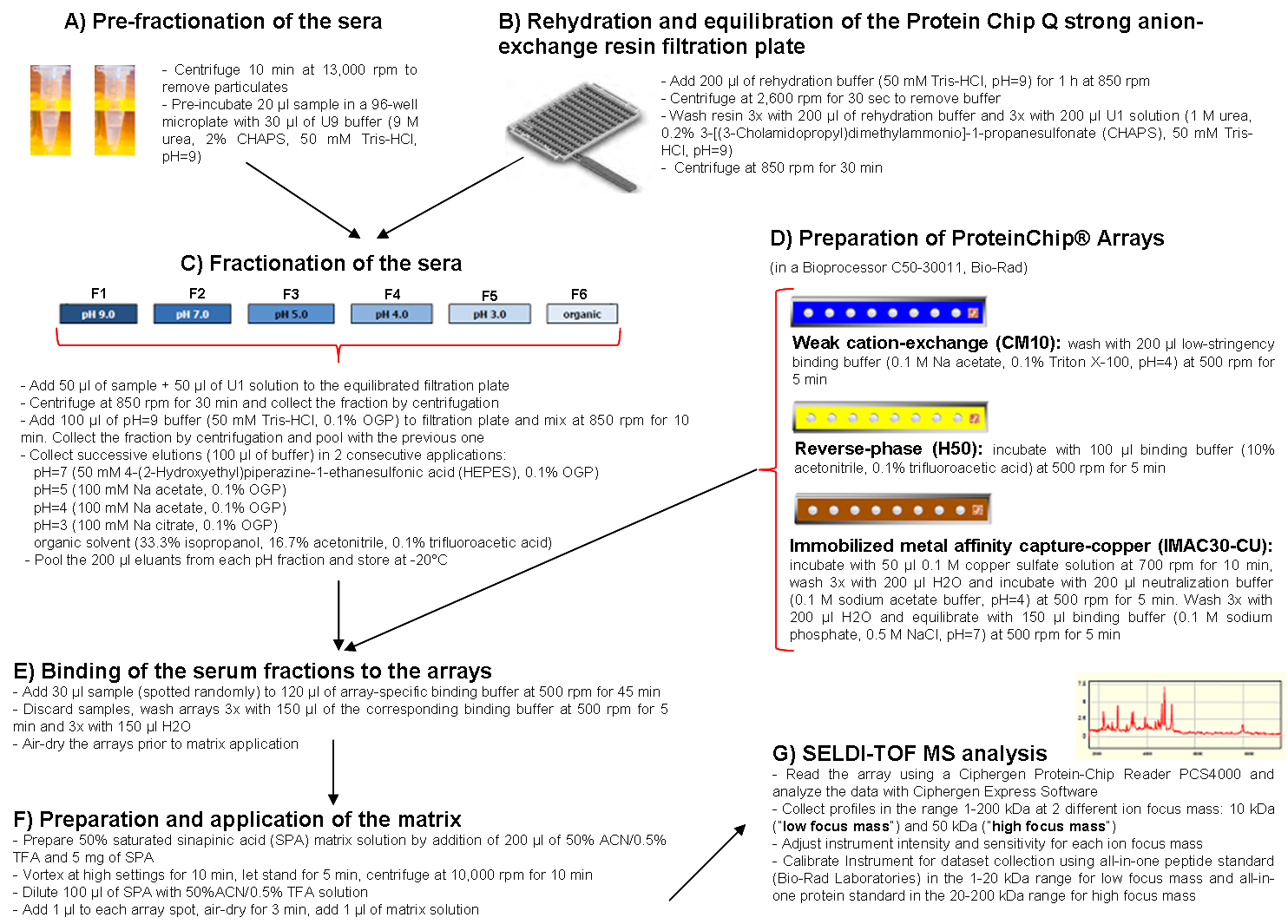

Supplement: Additional file 3 — Figure S1.Detailed protocol of the SELDI-TOF MS analysis. Schematic illustration of the protocol used for SELDI-TOF MS, which follows the manufacturer’s instruction manual with minor modifications (Bio-Rad Laboratories, ProteinChip® Serum Fractionation Kit manual). The protocol is divided in 7 main steps: A) Pre-fractionation of the sera; B) Rehydration and equilibration of the Protein Chip Q strong anion-exchange resin filtration plate; C) Fractionation of the sera; D) Preparation of ProteinChip® Arrays; E) Binding of the serum fractions to the arrays; F) Preparation and application of the matrix; and G) SELDI-TOF MS analysis. [file 1477-5956-10-48-S3.doc]
